# PARP-Inhibitor Treatment Prevents Hypertension Induced Cardiac Remodeling by Favorable Modulation of Heat Shock Proteins, Akt-1/GSK-3β and Several PKC Isoforms

**DOI:** 10.1371/journal.pone.0102148

**Published:** 2014-07-11

**Authors:** Laszlo Deres, Eva Bartha, Anita Palfi, Krisztian Eros, Adam Riba, Janos Lantos, Tamas Kalai, Kalman Hideg, Balazs Sumegi, Ferenc Gallyas, Kalman Toth, Robert Halmosi

**Affiliations:** 1 First Department of Medicine, Division of Cardiology, University of Pécs, Pécs, Hungary; 2 Szentagothai Janos Research Center, University of Pécs, Medical School, Pécs, Hungary; 3 Department of Surgical Research and Techniques, University of Pécs, Pécs, Hungary; 4 Department of Organic and Medicinal Chemistry, University of Pécs, Pécs, Hungary; 5 Department of Biochemistry and Medical Chemistry, Medical School, University of Pécs, Pécs, Hungary; 6 MTA-PTE Nuclear-Mitochondrial Interactions Research Group, Pécs, Hungary; University of Otago, New Zealand

## Abstract

Spontaneously hypertensive rat (SHR) is a suitable model for studies of the complications of hypertension. It is known that activation of poly(ADP-ribose) polymerase enzyme (PARP) plays an important role in the development of postinfarction as well as long-term hypertension induced heart failure. In this study, we examined whether PARP-inhibitor (L-2286) treatment could prevent the development of hypertensive cardiopathy in SHRs. 6-week-old SHR animals were treated with L-2286 (SHR-L group) or placebo (SHR-C group) for 24 weeks. Wistar-Kyoto rats were used as aged-matched, normotensive controls (WKY group). Echocardiography was performed, brain-derived natriuretic peptide (BNP) activity and blood pressure were determined at the end of the study. We detected the extent of fibrotic areas. The amount of heat-shock proteins (Hsps) and the phosphorylation state of Akt-1^Ser473^, glycogen synthase kinase (GSK)-3β^Ser9^, forkhead transcription factor (FKHR)^Ser256^, mitogen activated protein kinases (MAPKs), and protein kinase C (PKC) isoenzymes were monitored. The elevated blood pressure in SHRs was not influenced by PARP-inhibitor treatment. Systolic left ventricular function and BNP activity did not differ among the three groups. L-2286 treatment decreased the marked left ventricular (LV) hypertrophy which was developed in SHRs. Interstitial collagen deposition was also decreased by L-2286 treatment. The phosphorylation of extracellular signal-regulated kinase (ERK)1/2^Thr183-Tyr185^, Akt-1^Ser473^, GSK-3β^Ser9^, FKHR^Ser256^, and PKC ε^Ser729^ and the level of Hsp90 were increased, while the activity of PKC α/βII^Thr638/641^, ζ/λ^410/403^ were mitigated by L-2286 administration. We could detect signs of LV hypertrophy without congestive heart failure in SHR groups. This alteration was prevented by PARP inhibition. Our results suggest that PARP-inhibitor treatment has protective effect already in the early stage of hypertensive myocardial remodeling.

## Introduction

Left ventricular hypertrophy (LVH) represents the heart's response to increased biomechanical stress such as arterial hypertension or valvular heart disease. Cardiac hypertrophy has traditionally been considered a compensatory mechanism required to normalize wall tension and to maintain cardiac output. However, recent clinical studies as well as several animal models have shown that cardiac hypertrophy is rather a maladaptive process, ultimately leading to heart failure (HF) and sudden cardiac death independent of the underlying cause of hypertrophy [1].

Both physiologic and pathologic stimulation-induced cellular adaptations of the heart are typically initiated by stress-responsive signaling pathways, which serve as central transducers of cardiac hypertrophic growth and/or ventricular dilation. These signaling pathways include extracellular signal-regulated protein kinases (ERK), p38 mitogen-activated protein kinases (p38-MAPK), c-Jun NH_2_-terminal kinases (JNK) and several protein kinase C (PKC) isoforms [2]. These pathways and the Akt-1/glycogen synthase kinase-3β (GSK-3β) signaling cascade have all been demonstrated to alter their activation state in response to hypertrophic stimuli, and may therefore contribute to myocardial remodeling [3].

The poly(ADP-ribose) polymerase (PARP) enzyme becomes activated in response to DNA single-strand breaks that can be excessive as a response to free radicals and oxidative cell damage. PARP is an energy-consuming enzyme that transfers ADP-ribose to nuclear proteins. As a result of this process, the intracellular NAD^+^ and ATP levels decrease remarkably resulting in cell dysfunction and cell death via the necrotic route. Therefore, PARP-activation contributes to the pathogenesis of various cardiovascular diseases including endothelial dysfunction, ischemia-reperfusion injury and myocardial infarction, as well as HF. Several studies reported that endothelial dysfunction associated with hypertension also depends on PARP activity and can be prevented by its pharmacological inhibition [4], [5].

**Figure 1:**
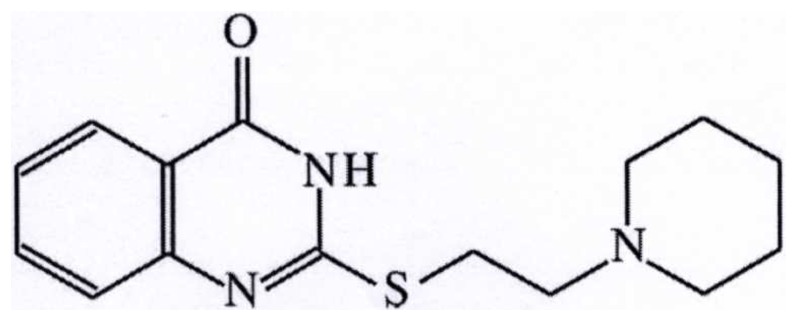
Chemical structure of L-2286 (2-[(2-Piperidine-1-ylethyl)thio]quinazolin-4(3H)-one).

It has been shown previously that our experimental agent, an isoquinoline derivative PARP-inhibitor, L-2286 (Fig. 1) had a beneficial effect against oxidative cell damage, against ischemia-reperfusion injury and the development of postinfarction or long-term high blood pressure-induced heart failure. Although the molecule have a slight scavenger characteristic, its forementioned effects were mediated mainly by influencing the Akt-1/GSK-3β, MAPK and PKC signal transduction factors [3], [6], [7].

Hypertension in spontaneously hypertensive rat (SHR) is similar to that of human in numerous ways such as the occurence of long-term, stable LVH followed by a transition to HF [8], [9], [10]. It makes SHR a useful tool for studying the development of LVH [9] and HF, well separated from each other in time. Therefore, our present study aimed to clarify whether pharmacological PARP-inhibition has protective effect in an SHR model against the development of the early stage of hypertensive cardiac remodeling.

## Materials and Methods

### Ethics Statement

The investigation conforms to the Guide for the Care and Use of Laboratory Animals published by the U.S. National Insitutes of Health (NIH Publication No. 85-23, revised 1996), and was approved by the Animal Research Review Committee of the University of Pecs, Medical School.

### Experimental protocol

Six weeks old male WKY-strain Wistar Kyoto and spontaneously hypertensive rats (Charles River Laboratories, Budapest, Hungary) were used. Animals were kept under standard conditions throughout the experiment; 12 h light-dark cycle, water and rat chow provided ad libitum. SHRs were randomly divided into two groups; SHR-L and SHR-C. SHR-L group was treated with L-2286 (2-[(2-piperidin-1-ylethyl)thio]quinazolin-4(3*H*)-one), a water-soluble PARP-inhibitor (5 mg/b.w. in kg/day, n = 12), while SHR-C group received only placebo (n = 11, SHR-C) p. os for 24 weeks [11], [12]. WKY rats were used as age-matched controls (n = 10). Dosage of L-2286 administered in drinking water was based on preliminary data about the volume of daily consumption [3], [6]. At the beginning and at the end of the 24-week-long period, echocardiographic measurements were performed. Invasive blood pressure measurements were carried out on 3 rats of each group at the end of the study. These rats were anesthetized with ketamine hydrochloride (Richter Gedeon Ltd., Budapest, Hungary) intraperitoneally and a polyethylene catheter (Portex, London, UK) was inserted into their left femoral artery. Systolic, diastolic and mean arterial blood pressure was determined by CardioMed System CM-2005 (Medi-Stim AS, Oslo, Norway). Animals were euthanized with an overdose of ketamine hydrochloride intraperitoneally and heparinized with sodium heparin (100 IU/rat i.p., Biochemie GmbH, Kundl, Austria). After the sacrifice, blood was collected to determine the concentration of plasma brain-derived natriuretic peptide (BNP), and hearts were removed, the atria and great vessels were trimmed from the ventricles and weight of the ventricles was measured, which was then normalized to body mass (index of cardiac hypertrophy). The lung wet weight-to-dry weight ratio (an index of pulmonary congestion) was also measured in 7–9 experimental animals [3]. Hearts were freeze-clamped and were stored at −70°C or fixed in 10% formalin. In order to detect the extent of fibrotic areas, histologic samples were stained with Masson's trichrome. The phosphorylation state of Akt-1/GSK-3β, MAPK and PKC signaling molecules were monitored by Western blotting.

### Determination of plasma B-type natriuretic peptide

Blood samples were collected into Lavender Vacutainer tubes containing EDTA and aprotinin (0.6 IU/ml of blood), and were centrifuged at 1600 g for 15 minutes at 4°C to separate the plasma. Supernatants were collected and kept at −70°C. BNP-45 were determined by enzyme immunoassay method as the manufacturer proposed (BNP-45, Rat EIA Kit, Phoenix Pharmaceuticals Inc., CA, USA).

### Histology

Ventricles fixed in formalin were embedded in paraffin, and 5 µm thick sections were cut from base to apex. Sectiones were stained with Masson's trichrome staining to detect the interstitial fibrosis, and quantified by the NIH ImageJ image processing program as described previously [3].

### Western blot analysis

Fifty milligrams of heart samples were homogenized in ice-cold 50 mM Tris buffer, pH 8.0 containing protease inhibitor cocktail 1∶100, and 50 mM sodium vanadate (Sigma-Aldrich Co., Budapest, Hungary), and were harvested in 2x concentrated sodium dodecyl sulphate (SDS)-polyacrylamide gel electrophoresis sample buffer. Proteins were separated on 10% or 12% SDS-polyacrylamide gel and transferred to nitrocellulose membranes. After blocking (2 h with 3% nonfat milk in Tris-buffered saline), membranes were probed overnight at 4°C with primary antibodies recognizing the following antigenes: phospho-specific Akt-1/protein kinase B-α Ser^473^ (1∶1000), Actin (1∶10000), phospho-specific glycogen synthase kinase (GSK)-3β Ser^9^ (1∶1000), phospho-specific extracellular signal-regulated kinase (ERK 1/2) Thr^202^-Tyr^204^ (1∶1000), phospho-specific p38 mitogen-activated protein kinase (p38-MAPK) Thr^180^-Gly-Tyr^182^ (1∶1000), phospho-specific c-Jun N-terminal kinase (JNK) Thr^183^-Tyr^185^ (1∶1000), phospho-specific protein kinase C (PKC) (pan) βII Ser^660^ (1∶1000), phospho-specific protein kinase C α/βII (PKC α/βII) Thr^638/641^ (1∶1000), phospho-specific protein kinase C δ (PKC δ) Thr^505^ (1∶1000), phospho-specific protein kinase C ζ/λ (PKC ζ/λ) Thr^410/403^ (1∶1000), phospho-specific protein kinase C ε (PKC ε) Ser^729^ (1∶1000), anti-poly(ADP-ribose) (anti-PAR, 1∶5000), phospho-Foxo1A (forkhead transcription factor, FKHR Ser^256^, 1∶1000), Heat shock protein 72 (Hsp72, 1∶20000), Heat shock protein 90 (Hsp90, 1∶1000). Antibodies were purchased from Cell Signaling Technology (Beverly, MA, USA) except from actin, which was bought from Sigma-Aldrich Co, (Budapest, Hungary), phospho-specific PKC ε, which was purchased from Upstate (London, UK), anti-PAR, which was purchased from Alexis Biotechnology (London, UK), Hsp90, which was bought from Santa Cruz Biotechnology (Wembley, UK), Hsp72, which was purchased from StressGene Biomol GmbH (Hamburg, Germany). Membranes were washed six times for 5 min in Tris-buffered saline, pH 7.5 containing 0.2% Tween before addition of goat anti-rabbit horseradish peroxidase-conjugated secondary antibody (1∶3000 dilution, Bio-Rad, Budapest, Hungary). The antibody-antigen complexes were visualized by means of enhanced chemiluminescence. After scanning, results were quantified by NIH ImageJ program. Pixel densities of bands were normalized to that of the loading controls.

### Noninvasive evaluation of cardiac functions and dimensions

At the start of the experiment, all animals were examined by echocardiography to exclude rats with any heart abnormalities. Transthoracic two-dimensional echocardiography was performed under inhalation anesthesia at the beginning of the experiment and on the day of sacrifice. Rats were lightly anesthetized with a mixture of 1.5% isoflurane (Forane, Abbott Laboratories, Hungary) and 98.5% oxygen. The chest of animals was shaved, acoustic coupling gel was applied and warming pad was used to maintain normothermia. Rats were imaged in the left lateral decubitus position. Cardiac dimensions and functions were measured from short- and long-axis views at the mid-papillary level by a VEVO 770 high-resolution ultrasound imaging system (VisualSonics, Toronto, Canada) equipped with a 25 MHz transducer. LV fractional shortening (FS), ejection fraction (EF), LV end-diastolic volume (LVEDV), LV end-systolic volume (LVESV), and the thickness of septum and posterior wall were determined. FS (%) was calculated by 100x((LVID_d_-LVID_s_)/LVID_d_) (LVID: LV inside dimension; d: diastolic; s: systolic), EF (%) was calculated by 100x((LVEDV-LVESV)/LVEDV), relative wall thickness (RWT) was calculated by (PW thickness + interventricular septal thickness)/LVID_d_.

### Statistical analysis

All data are expressed as mean±SEM. First of all the homogeneity of the groups was tested by F-test (Levene's test). There were no significant differences among the groups. Comparisons among groups were made using one-way ANOVA (SPSS for Windows 11.0). For post hoc comparison Bonferroni test was chosen. Values of p<0.05 were considered statistically significant.

## Results

Effect of L-2286 on normotensive WKY rats was also examined, but the investigated parameters did not differ significantly from the non-treated WKY animals. Therefore, data of L-2286 treated WKY rats were not shown to avoid unnecessary redundancies.

### Effect of PARP inhibition on gravimetric parameters of spontaneously hypertensive rats

Body weights did not differ significantly among the three groups (WKY: 71.01±0.11 g, SHR-C: 72.03±2.36 g, SHR-L: 69.92±3.21 g, 6-week-old rats) at the beginning of our study. However, at the end of the 24-week-long treatment period, body weights of WKY group were significantly higher than those of SHR-C and SHR-L groups (WKY: 392.7±14.01 g, SHR-C: 323.8±11.27 g, SHR-L: 321.9±6.84 g, p<0.01 WKY vs. SHR groups, 30-week-old rats). The degree of myocardial hypertrophy was determined by ventricular weight to body weight ratio (WV/BW, mg/g). This parameter was significantly increased in SHR groups compared to the WKY group (WV/BW: WKY: 2.95±0.17, SHR-C: 4.48±0.12, SHR-L: 3.85±0.15, p<0.05 WKY vs. SHR groups). Similar results were obtained in case of weights of ventricles (WV, WKY: 1.16±0.17 g, SHR-C: 1.45±0.18 g, SHR-L: 1.24±0.24 g, p<0.05 WKY vs. SHR groups). The WV and WV/BW ratios were significantly decreased by L-2286 treatment (p<0.05 SHR-L vs. SHR-C). The lung wet weight-to-dry weight ratio was not elevated significantly in SHR-C and SHR-L compared to WKY groups (Table 1). All these results indicate the presence of cardiac hypertrophy without congestive heart failure in the SHR-C group that was ameliorated in the SHR-L group.

**Table 1:** Effect of L-2286 treatment on gravimetric parameters and on plasma BNP in SHR.

	WKY	SHR-C	SHR-L
SAP^30w^, (mmHg)	129±7	192±9^b^	186±5^b^
DAP^30w^, (mmHg)	89±5	127±8^b^	125±4^b^
MAP^30w^, (mmHg)	103±7	149±5^b^	146±7^b^
BW^6w^ (g)	71.01±1.89	72.02±2.36	69.9±3.21
BW (g)	393±14.01	323.8±11.27^a^	321.86±6.8^a^^,^ c
WV (g)	1.16±0.17	1.45±0.18^b^	1.24±0.24^b^^,^ c
WV/BW (mg/g)	2.95±0.17	4.48±0.12^b^	3.85±0.15^b^^,^ c
Lung wet weight/dry weight	4.84±0.92	4.79±0.84	4.77±0.99
p-BNP (ng/ml)	2.19±0.011	2.33±0.034	2.31±0.031

WKY: normotensive age-matched control rats, n = 7, SHR-C: SHR age-matched control rats, n = 8, SHR-L: SHR treated with L-2286 for 24 weeks, n = 9. SAP, DAP, MAP^30w^: systolic, diastolic and mean arterial blood pressure at 30-week-old age (n = 3 from each group). BW^6w^: body weight of 6-week-old rats, BW: body weight, WV: weights of ventricles, BNP: plasma b-type natriuretic peptide. Values are means±S.E.M.

a <0.01 (vs. WKY group),

b <0.05 (vs. WKY group),

c <0.05 (vs. SHR-C).

### L-2286 treatment did not influence the levels of plasma BNP and blood pressure

Slightly elevated plasma BNP levels were found both in SHR-C and SHR-L groups (not significant vs. WKY group). Although plasma BNP level was a little higher in SHR-C group than in SHR-L group, this difference was also not statistically significant (Table 1). In both SHR groups, blood pressure was significantly elevated compared to the WKY group (p<0.05). L-2286 treatment did not decrease significantly the elevated blood pressure (Table 1).

### L-2286 decreased the interstitial collagen deposition in the myocardium

**Figure 2:**
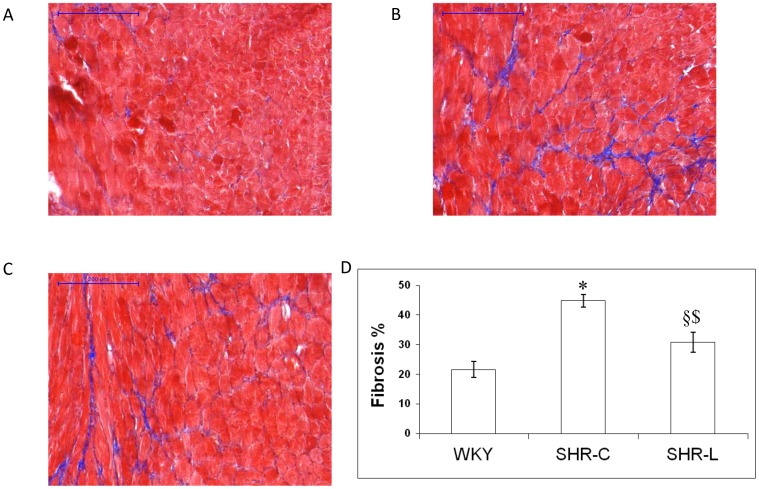
L-2286 treatment decreased the deposition of interstitial collagen. Sections stained with Masson's trichrome (n = 5). Scale bars mean 200 µm. Magnifications 10-fold. WKY (A): normotensive age-matched control rats. SHR-C (B): 30 week-old spontaneously hypertensive rats, SHR-L (C): 30 week-old spontaneously hypertensive rats treated with L-2286 for 24 week. D: Denzitometric evaluation of the sections is shown. ^*^p<0.01 vs. WKY, ^§^p<0.05 vs. WKY, ^$^p<0.05 vs. SHR-C.

Histological analysis revealed slight interstitial collagen deposition in the WKY group. Chronic high blood pressure caused significantly higher collagen deposition in SHR-C rats that was significantly diminished (p<0.05) in the SHR-L group (Fig. 2).

### PARP inhibition decreased the left ventricular hypertrophy in spontaneously hypertensive rats

At the beginning of the study the echocardiographic parameters of the three groups did not differ significantly from each other (Table 2). At the age of 30 weeks there was no significant difference in LV systolic functions (EF and FS) between the WKY and SHR groups. Heart rate did not differ significantly during the anesthesia among the groups. LVESV and LVEDV were increased significantly in SHRs (p<0.05 WKY vs. SHR-C and SHR-L), and these unfavorable alterations were not reduced by L-2286 treatment. The thickness of the septum, and the posterior wall and the relative wall thickness were also increased in SHR groups (indicating the presence of ventricular hypertrophy) comparing to the WKY group (p<0.05), and these parameters could be significantly reduced by the administration of L-2286 (p<0.05 SHR-C vs. SHR-L group) (Table 3).

**Table 2:** L-2286 treatment moderately influenced the echocardiographic parameters in 6 weeks old SHRs.

	WKY	SHR-C	SHR-L
EF (%)^6w^	67.26±0.525	68.4±1.77	68.23±1.81
FS^6w^	38.63±4.47	38.03±5.52	39.35±4.15
LVEDV^6w^ (ml)	147.27±13.88	149.56±16.78	149.11±14.43
LVESV^6w^ (ml)	46.63±4.47	48.03±5.52	47.35±5.45
Septum^6w^ (mm)	1.2±0.07	1.18±0.05	1.17±0.12
PW^6w^ (mm)	1.19±0.07	1.16±0.067	1.14±0.04
LV mass^6w^ (uncorrected)	344.14±35.49	351.66±36.23	354.77±33.23

WKY: normotensive age-matched control rats, n = 7, SHR-C: SHR age-matched control rats, n = 8, SHR-L: n = 9, SHR treated with L-2286 for 24 weeks.EF^6w^: ejection fraction, FS^6w^: fractional shortening, LVEDV^6w^: left ventricular (LV) end-diastolic volume, LVESV^6w^: LV end-systolic volume, Septum^6w^: thickness of septum, PW^6w^: thickness of posterior wall, LV mass^6w^: weights of LVs.

**Table 3:** L-2286 treatment moderately influenced the echocardiographic parameters in 30 weeks old SHRs.

	WKY	SHR-C	SHR-L
EF (%)^30w^	69.1±2.4	68.72±2.1	69.01±3.2
FS^30w^	39.8±1.9	39.04±1.85	40.57±2.66
LVEDV^30w^ (ml)	279.18±18.18	335.87±10.36^a^	326.94±9.18^a^
LVESV^30w^ (ml)	85.77±8.56	96.85±10.36^a^	99.81±11.85^a^
Septum^30w^ (mm)	1.43±0.04	1.93±0.04^a^	1.79±0.05^a^^,^ b
PW^30w^ (mm)	1.54±0.08	2.15±0.12^a^	1.87±0.03^a^^,^ b
RWT^30w^	0.38±0.05	0.504±0.02^a^	0.445±0.012^a^^,^ b
LV mass^30w^ (uncorrected)	1002.81±59.5	1370.35±79.87^a^	1121.13±53.23^a^^,^ b
LV mass^30^/BW^30^ (mg/g)	2.73±0.7	4.23±0.8^a^	3.70±0.3^a^^,^ b

WKY: normotensive age-matched control rats, n = 7, SHR-C: SHR age-matched control rats, n = 8, SHR-L: n = 9, SHR treated with L-2286 for 24 weeks.EF^30w^: ejection fraction, F^30w^: fractional shortening, LVEDV^30w^: left ventricular (LV) end-diastolic volume, LVESV^30w^: LV end-systolic volume, Septum^30w^: thickness of septum, PW^30w^: thickness of posterior wall, RWT^30w^: relative wall thickness, LV mass^30w^: weights of LVs. Values are mean±S.E.M.

a p<0.05 (vs. WKY group),

b p<0.05 (vs. SHR-C group).

### Effect of L-2286 treatment on poly-ADP-ribosylation as well as on the phosphorylation state of Akt-1^Ser473^/GSK-3β^Ser9^ and FKHR^Ser256^

**Figure 3:**
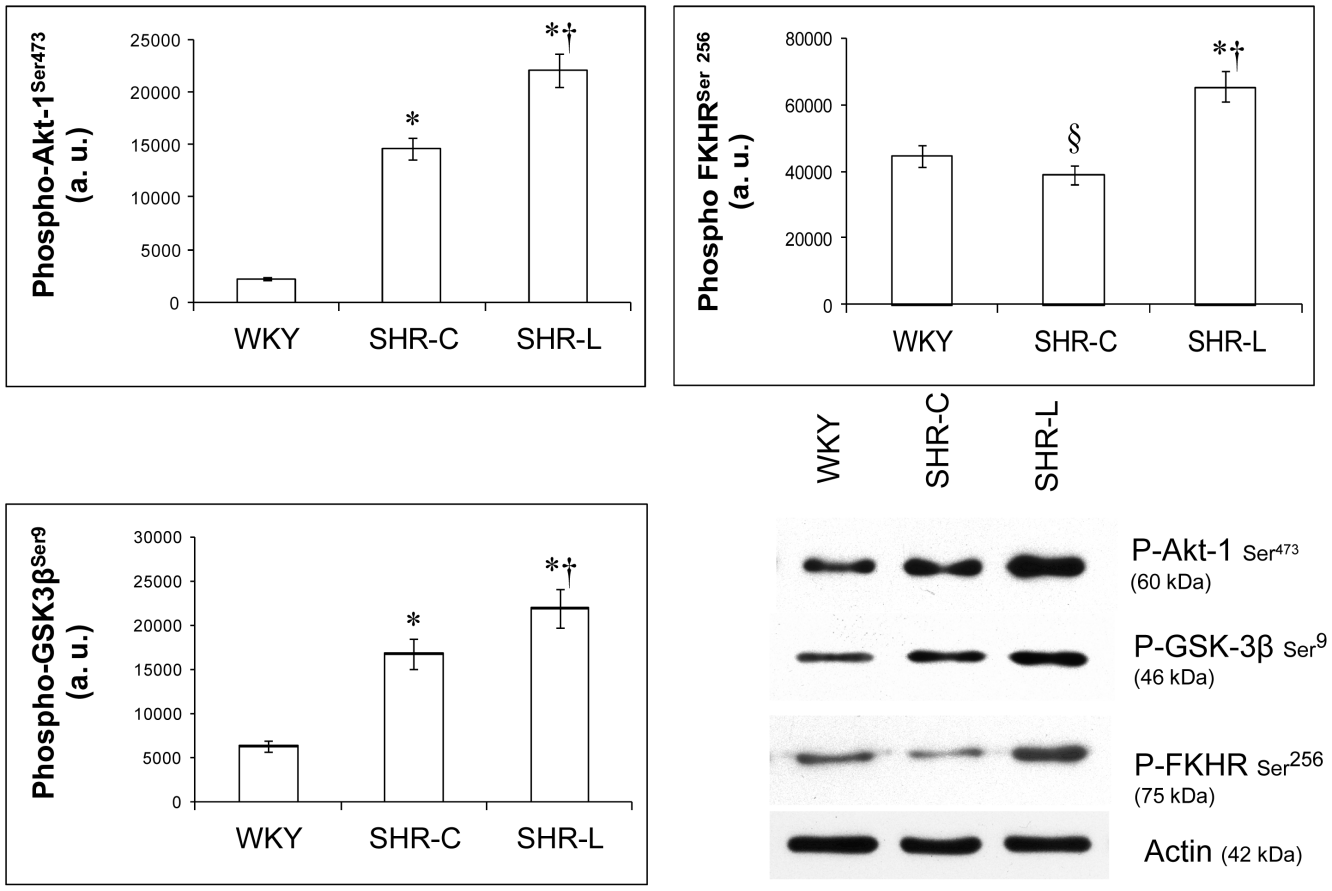
Effect of L-2286 treatment on Akt-1^Ser473^/GSK-3β^Ser9^, FKHR^Ser256^ pathway. Representative Western blot analysis of Akt-1^Ser473^, GSK-3β^Ser9^, FKHR^Ser256^ phosphorylation and densitometric evaluation is shown (n = 4). Actin was used as loading control. Values are means±S.E.M. WKY: normotensive age-matched control rats. SHR-C: 30 week-old spontaneously hypertensive rats, SHR-L: 30 week-old spontaneously hypertensive rats treated with L-2286 for 24 weeks. ^*^p<0.01 vs. WKY, ^†^p<0.01 vs. SHR-C, ^§^p<0.05 vs. WKY.

Akt-1^Ser473^ was moderately phosphorylated in WKY group. In SHR-C group, the phosphorylation of Akt-1^Ser473^ was more pronounced (p<0.01 vs. WKY). Moreover, in SHR-L rats the L-2286 treatment caused further elevation in Akt-1^Ser473^ phosphorylation (p<0.01 vs. WKY and SHR-C groups) (Fig. 3). The same result was obtained in the case of GSK-3β^Ser9^ phosphorylation (Fig. 3).

**Figure 4:**
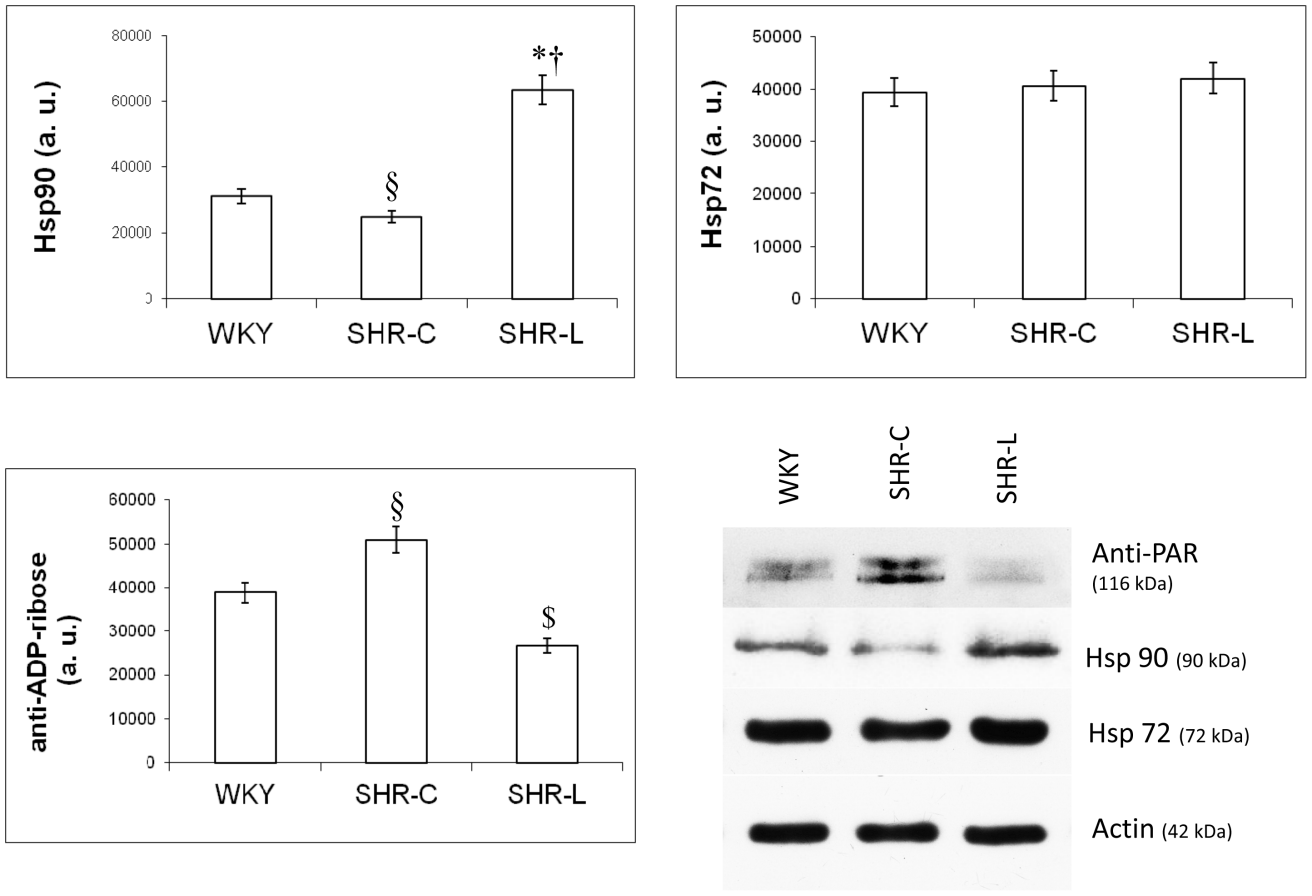
Effect of L-2286 treatment on the level of Hsp72, 90 and poly(ADP-ribos)ylation. Representative Western-blot analysis of Hsp72, 90, anti-PAR and densitometric evaluations are shown (n = 4). Actin was used as loading control. Values are means±S.E.M. WKY: normotensive age-matched control rats. SHR-C: 30 week-old spontaneously hypertensive rats, SHR-L: 30 week-old spontaneously hypertensive rats treated with L-2286 for 24 weeks. ^*^p<0.01 vs. WKY, ^†^p<0.01 v.s SHR-C, ^§^p<0.05 vs. WKY, ^$^p<0.05 vs. SHR-C.

Another target protein of Akt-1^Ser473^ (besides GSK3β^Ser9^) is FKHR^Ser256^. Consistently with the result of Akt-1^Ser473^ phosphorylation, the strongest phosphorylation (therefore inhibition) could be observed in SHR-L group (p<0.01 vs. SHR-C and WKY). The lowest phosphorylation and therefore the highest activity of FKHR was seen in SHR-C group (p<0.05 vs. WKY, Fig. 3). To detect the effectivity of L-2286, the ADP-ribosylation of the samples were analysed by Western-blot. The lowest degree of ADP-ribosylation was present in SHR-L group, and the most pronounced ADP-ribosylation was seen in SHR-C group (p<0.05 vs. WKY) (Fig. 4).

### Effect of L-2286 on the amount of Hsp72 and 90

There was no significant difference among the three groups in the level of Hsp72. On the other hand, the level of Hsp90 was elevated in SHR-L group compared to WKY and SHR-C groups (p<0.01 SHR-L vs. WKY or SHR-C groups), and the lowest amount of this protein was present in WKY samples (Fig. 4).

### Effect of L-2286 administration on MAPKs

**Figure 5:**
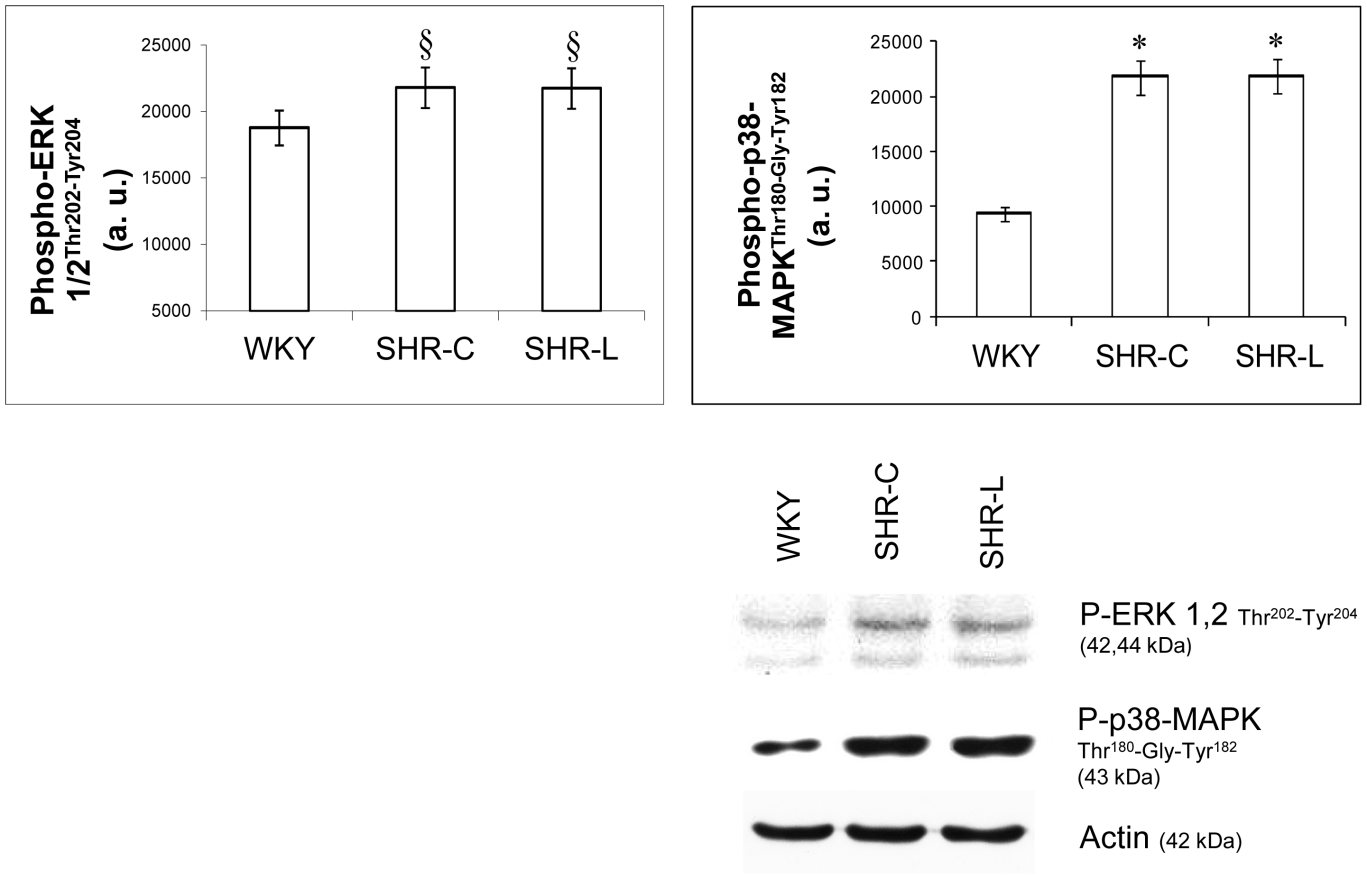
Effect of L-2286 on the phosphorylation state of MAPK pathway. Representative Western blot analysis of ERK 1/2^Thr183-Tyr185^, and p38-MAPK^Thr180-Gly-Tyr182^ phosphorylation and densitometric evaluation is shown (n = 4). Actin was used as loading control. Values are means±S.E.M. WKY: normotensive age-matched control rats. SHR-C: 30 week-old spontaneously hypertensive rats, SHR-L: 30 week-old spontaneously hypertensive rats treated with L-2286 for 24 weeks. ^§^p<0.05 vs. WKY ^*^p<0.01 vs. WKY.

Phosphorylation of p38-MAPK^Thr180-Gly-Tyr182^, ERK 1/2^Thr183-Tyr185^ and JNK was the lowest in the WKY group compared to SHR-C and SHR-L groups (p38-MAPK^Thr180-Gly-Tyr182^: p<0.01 vs. SHR groups, ERK 1/2: p<0.05 vs. SHR groups, JNK: p<0.05 vs. SHR groups). In the case of p38-MAPK^Thr180-Gly-Tyr182^, ERK 1/2^Thr183-Tyr185^ and JNK, their phosphorylation was elevated in both SHR-C and SHR-L groups, but there were no significant differences between the two SHR groups (Fig. 5, JNK: data not shown).

### Influence of L-2286 treatment on the phosphorylation state of several PKC isoforms

**Figure 6:**
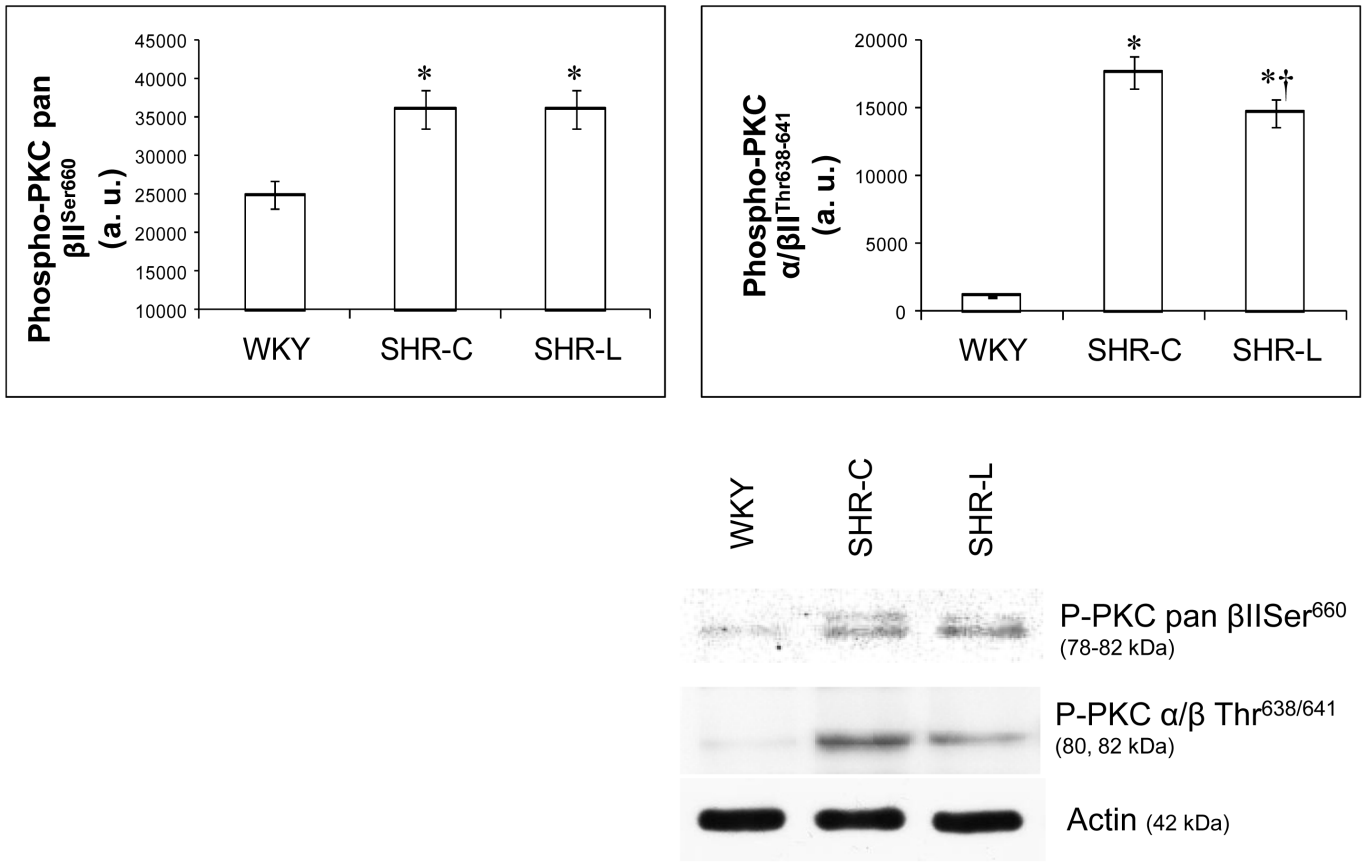
Effect of L-2286 administration on the activity of PKC isoenzymes. Representative Western blot analysis of PKC pan βII^Ser660^ and PKC α/βII^Thr638/641^ phosphorylation and densitometric evaluations are shown (n = 4). Values are means±S.E.M. WKY: normotensive age-matched control rats. SHR-C: 30 week-old spontaneously hypertensive rats, SHR-L: 30 week-old spontaneously hypertensive rats treated with L-2286 for 24 weeks. ^*^p<0.01 vs. WKY, ^†^p<0.01 vs. SHR-C.

The overall (pan) phosphorylation of PKC (pan βII^Ser660^) was low in the WKY group and became significantly higher in SHR-C and SHR-L groups (p<0.01 WKY vs. SHR groups). Administration of L-2286 could not affect the phosphorylation state of PKC pan βII Ser^660^ in SHR-L group compared to the SHR-C group (Fig. 6).

**Figure 7:**
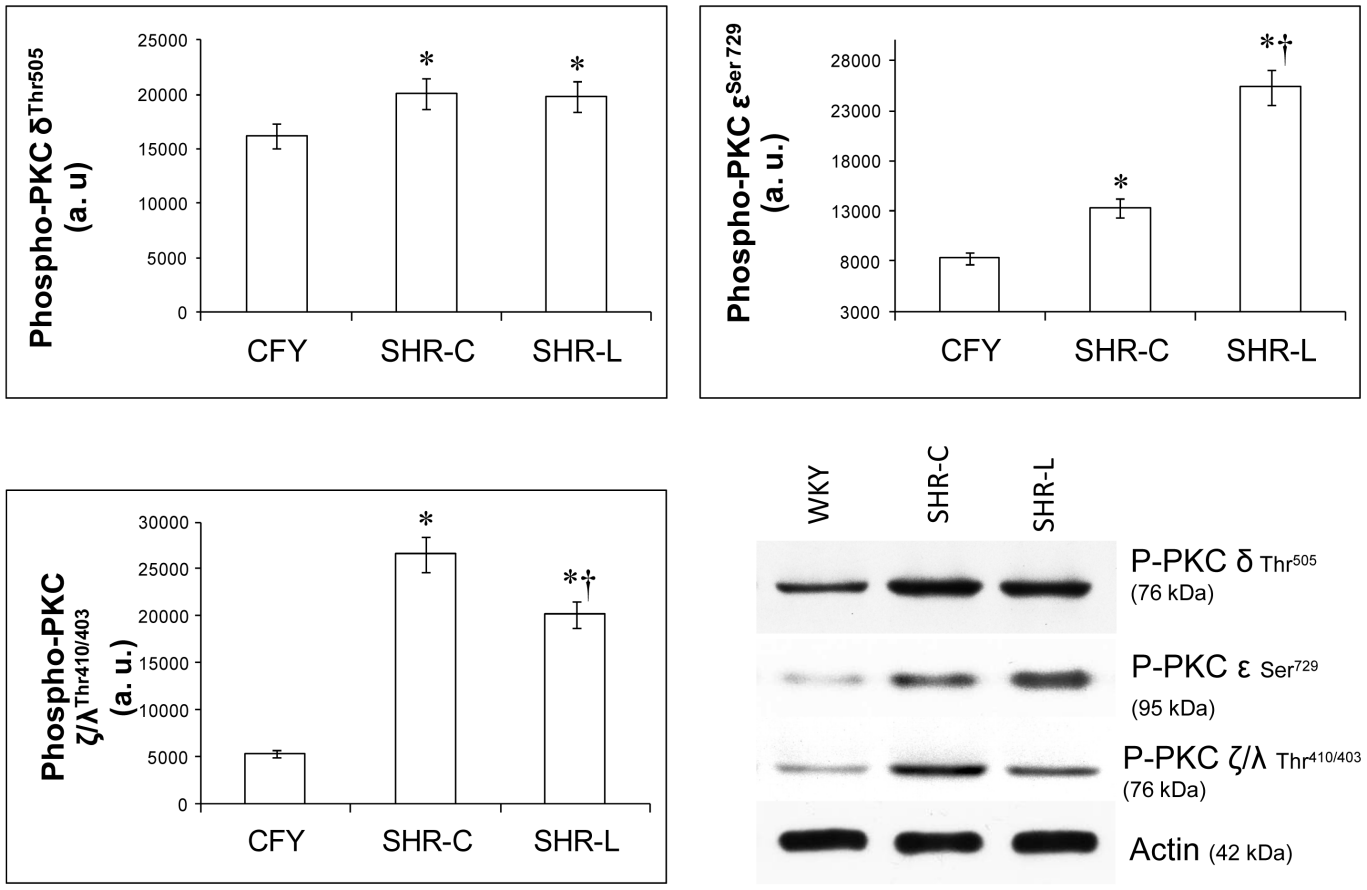
Effect of L-2286 administration of PKC isoenzymes. Representative Western blot analysis of PKC δ^Thr505^, ε^Ser729^ and ζ/λ^Thr410/403^ phosphorylation and densitometric evaluation is shown (n = 4). Actin was used as loading control. Values are means±S.E.M. WKY: normotensive age-matched control rats. SHR-C: 30 week-old spontaneously hypertensive rats, SHR-L: 30 week-old spontaneously hypertensive rats treated with L-2286 for 24 weeks. ^*^p<0.01 vs. WKY, ^†^p<0.01 vs. SHR-C.

The lowest phosphorylation could be observed in the WKY group in case of PKC α/βII^Thr638/641^, δ^Thr505^, ζ/λ^Thr410/403^ and ε^Ser729^ (p<0.01 vs. SHR groups). As PKC ζ antibody, we used a combined antibody (i.e. PKC ζ/λ Thr^410/403^), which did not discriminate between PKC ζ and λ; PKC λ being structurally highly homologous to PKC ζ in the COOH-terminal end of the molecule [12]. L-2286 treatment decreased significantly the phosphorylation of PKC α/βII ^Thr638/641^ and ζ, while it could increase the phosphorylation of ε^Ser729^ (PKC α/βII ^Thr638/641^, ζ, ε^Ser729^: p<0.01, SHR-L vs. SHR-C) (Fig. 6,7). In the case of PKC δ^Thr505^ there was no significant difference between the SHR groups (Fig. 7).

## Discussion

The major findings of this study are that chronic inhibition of nuclear PARP enzyme reduces excessive ADP-ribosylation of nuclear proteins, beneficially influences the intracellular signaling pathways and thus prevents the development of cardiac hypertrophy, which is an early consequence of hypertension. We used the SHR model that is a relevant animal model of essential hypertension in humans [13]. Our study began at a very early age (6-week-old) of SHRs, because at this age the blood pressure of animals is still normal and the hearts show no signs of remodeling. However, by the end of the study (30 weeks), marked signs of hypertensive cardiopathy develop in SHRs.

Previously, we have proved that PARP-inhibition could inhibit the transition of hypertensive cardiopathy to end-stage heart failure [6], but there is no data about the role of PAPR-inhibitors against the development of early consequences of hypertension.

Hypertension is a major risk factor for cardiovascular mortality and morbidity, and it is associated with left ventricular hypertrophy and diastolic dysfunction and later with systolic dysfunction and it can lead to heart failure. There is a strong correlation between left ventricular mass and the development of cardiovascular pathogies [14]. The development of long-term hypertension-induced myocardial remodeling can be explained by different mechanism in the literature, but generally, oxidative stress and abnormal signaling are considered as molecular basis of the disease. Peroxynitrite and other reactive species induce oxidative DNA damage and consequent activation of the nuclear enzyme PARP. In related animal models of the disease, pharmacological inhibition of PARP provides significant therapeutic benefits [15].

### PARP inhibition and gravimetric parameters in SHR

Significant LV hypertrophy develops by the age of 3 months in SHR animals but it is more often studied closer to 6 months of age [16]. In our SHR rats myocardial hypertrophy developed, as increased WV/BW ratio could be observed. We could not observe any obvious signs of HF, because BNP activity and the index of pulmonary congestion was not elevated compared to the WKY group.

### PARP inhibition and interstitial collagen deposition in SHR

Chronic hypertension leads to excessive collagen deposition (fibrosis) as part of the process of cardiovascular remodeling. In our previous studies, when SHR or postinfarcted animals exhibited overt heart failure, L-2286 also prevented interstitial fibrosis and adverse structural remodeling [3], [6]. In the present study, our results suggest that PARP inhibitor treatment can exert marked antifibrotic effect already in this early stage of hypertensive heart disease.

### PARP inhibition and echocardiographic parameters

In our experiment the systolic LV function was not decreased in SHR rats during the 24-week-long treatment. It is in accordance with several other studies [8], [9], [10] involving different experimental models of pressure overload-induced hypertrophy. During the development of hypertension, alterations in LV geometry may also occur as an adaptation to increased pressure overload. In hypertensive patients, LV geometry can be classified into four patterns on the basis of LV mass index and RWT and these patterns have been shown to be closely related to LV function and to patients' prognosis [17], [18], [19]. In this study, increased RWT and increased WV/BW were found, which indicates concentric LV hypertrophy [9]. L-2286 treatment decreased significantly the signs of left ventricular hypertrophy (wall thickness and RWT) even though the elevated blood pressure of SHR rats was not influenced by PARP inhibition.

### L-2286 treatment and the activity of Akt-1^Ser473^/GSK3β^Ser9^ and FKHR^Ser256^ pathway

Previous works demonstrated that PARP inhibitors can induce the phosphorylation and activation of Akt-1 in reperfused myocardium, thus raising the possibility that the protective effect of PARP inhibition can be mediated through the activation of the prosurvival phosphatidylinositol-3-kinase (PI3-kinase)/Akt-1 pathway [20]. Akt-1 is a key molecule in the signaling cascade of physiological hypertrophy [21]. Recent results demonstrate an important role of Akt/m-TOR signaling in cardiac angiogenesis, whose disruption contributes to the transition from hypertrophy to HF [1]. In our experiment, the phosphorylation of Akt-1^Ser473^ was far the lowest in WKY group and the highest in SHR-L group. Phosphorylation and therefore the inhibition of GSK-3β^Ser9^ and Foxo1 (FKHR) (downstream targets of Akt-1) [22], [23] were also determined. This showed the same pattern as the phosphorylation of Akt-1. The similar results were obtained in our studies using PARP inhibitors [20], [24] or by suppressing PARP-1 activation by siRNA technique [25]. These results may indicate that SHR-C animals tried to compensate for the adverse effects of chronic hypertension, but failed to do so. On the other hand, L-2286 treatment further elevated Akt activation that could, at least partially, account for the beneficial changes in the cardial remodeling of the SHR-L animals.

### L-2286 administration and levels of Hsp 72 and 90

Cellular stress leads to the expression of Hsp's [26]. They are known to protect the myocardium from the damaging effects of ischemia and reperfusion [27]. According to the results of Jiang et al [27] and Shinohara et al [28] the Hsp's can preserve the mitochondrial respiratory function and structure which are damaged in case of cell death. The flux of pro-apoptotic proteins can be induced by various stimuli, one of them is the decreased level of ATP. This can be induced by overactivation of PARP-1, which consumes too much ATP in certain pathologic conditions [29]. In case of Hsp90 in our study, the level of it was increased by long-term L-2286 treatment. Besides the activation of Akt-1^Ser473^, this can contribute to the cell survival in L-2286 treated rats. The level of Hsp72 was not influenced significantly by L-2286 administration in our investigation.

### L-2286 administration and MAPKs in young SHR

Previous works demonstrated that PARP inhibitors have a moderate effect on MAPKs in acute phase of myocardial infarction and in postinfarction heart failure [3], . MAPKs are ubiquitously expressed and their activation is observed in different heart diseases, including hypertrophic cardiomyopathy, dilated cardiomyopathy, and ischemic/reperfusion injury in human and animal models [30]. In our study, the phosphorylation of p38-MAPK^Thr180-Gly-Tyr182^, JNK and ERK 1/2^Thr183-Tyr185^ was elevated both in SHR-C and SHR-L groups. Our results are consistent with the results of Kacimi et Gerdes [31] using spontaneously hypertensive heart failure (SHHF) rats. L-2286 treatment did not influence the phosphorylation of p38-MAPK and JNK. The role of JNK and p38-MAPK-signaling in cardiac hypertrophy is not fully clarified [1]. However, both p38-MAPK and JNK transduction cascades have been implicated in the regulation of hypertrophic response as well as cardiomyopathy and HF [32]. JNK activity was not altered by L-2286 treatment in SHR animals, similarly to Hsp72 level. This result is in accordance with previous data [28] demonstrating that Hsp72 downregulates JNK by accelerating its dephosphorylation.

The elevated blood pressure may induced ERK activation [33]. Accordingly, activation of ERK1/2 was the lowest in WKY group, and was higher in SHR-C. Phosphorylation of ERK1/2 was not elevated by L-2286 administration in this study. The in vivo role of ERK in cardiac hypertrophy has been demonstrated in several genetically engineered animal models. Cardiac-specific expression of constitutively activated MEK1 promotes cardiac hypertrophy without compromised function or long-term animal survival, suggesting that activation of ERK activity promotes a compensated form of hypertrophy [30]. All these results suggest that MAPK activation did not participate significantly in mediating the adverse cardiac effects of chronic hypertension in our model.

### PARP inhibition and PKC pathways in young SHR

PARP inhibitors were found to affect PKC isoenzymes [3], [17]. The levels of all PKC isoforms increased in SHR groups compared to the WKY group in our study. Our results are in agreement with Koide et al. [34] using Dahl Salt-Sensitive rats in cardiac hypertrophy stage. Recent studies suggested that PKC is critically involved in the development of cardiac remodeling and HF. The data also suggest that individual PKC isoforms have different effects on cell signaling pathways, variously leading to changes in cardiac contractility, hypertrophic response and tolerance to myocardial ischaemia in the heart [34].

Activation of PKC pan βII^Ser660^ and δ^Thr505^ were not altered by L-2286 treatment, while activation of α/βII^Thr638/641^ and ζ/λ ^Thr410/403^ were attenuated and activation of ε^Ser729^ was augmented by L-2286. These alterations can mediate – at least partly – the favourable cardiovascular effects of L-2286, similarly as it was found in previous works [3], [20].

PKC α is the most extensively expressed among the myocardial PKC isoforms, and it is a key regulator of cardiomyocyte hypertrophic growth [35], [36]. PKC α was sufficient to stimulate cell hypertrophy [37]. Transgenic mice overexpressing PKC β2 exhibited cardiac hypertrophy and decreased LV performance; this depressed cardiac function improved after the administration of a PKC β-selective inhibitor [38]. Previous reports suggest that PKC α and β and PKC ζ/λ are involved in the development of cardiac hypertrophy and HF. Additionally, PKC ε plays a role in physiological hypertrophic responses [34], [39], has cardioprotective effect [40] and by interacting with Akt-1 and affecting Bcl-2 promote vascular cytoprotection [41]. Accordingly, in our study, both the activity of Akt-1^Ser473^ and PKCε^Ser729^ were elevated by L-2286 administration.

## Conclusions

**Figure 8:**
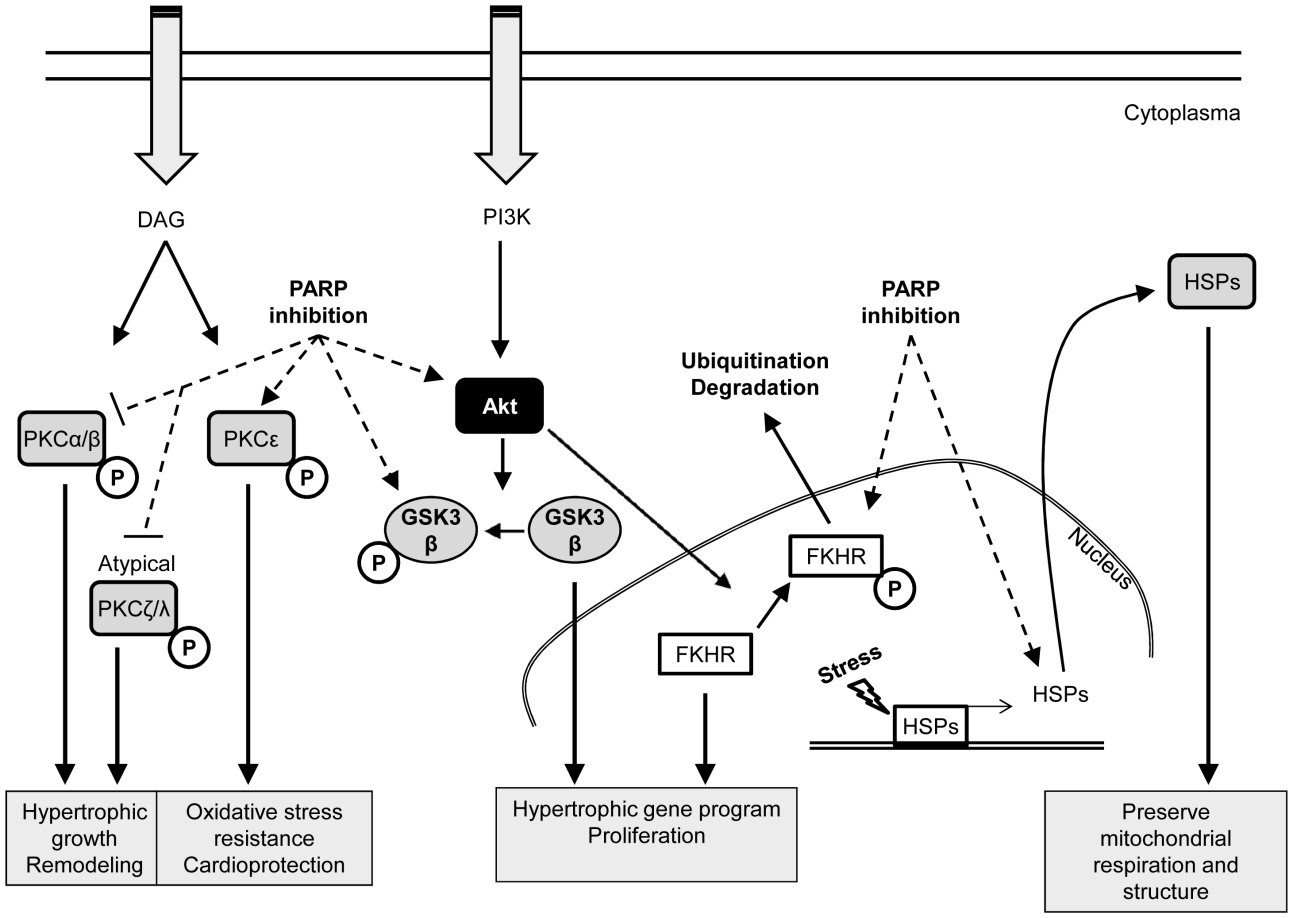
Summary of pathway alterations due to L-2286 treatment.

In our study, we examined the effect of a PARP inhibitor (L-2286) in SHR at the stage of LV hypertrophy. L-2286 exerted a beneficial effect on the progression of myocardial hypertrophy (thickness of PW and septum, RWT) and myocardial fibrosis. In the background of these changes, we did not observe any blood pressure lowering effect of PARP-inhibition. According to our results, PARP-inhibition can exert this antihypertrophic effect due to the activation of several prosurvival (especially Akt-1/GSK-3β, FKHR, PKCε and Hsp90) and the inhibition of prohypertrophic (PKC- α/βII, - ζ/λ) protein kinases (Fig.8).
